# Lipocalin-2 and Cerebral Stroke

**DOI:** 10.3389/fnmol.2022.850849

**Published:** 2022-04-12

**Authors:** Chao Luo, Shuai Zhou, Shi Yin, Lipeng Jian, Pengren Luo, Jigeng Dong, Erheng Liu

**Affiliations:** ^1^Department of Neurosurgery, The Affiliated Hospital of Kunming University of Science and Technology, Kunming, China; ^2^Department of Neurosurgery, The First People’s Hospital of Yunnan Province, Kunming, China

**Keywords:** cerebral stroke, lipocalin-2 (LCN-2), blood brain barrier, central nervous system, secondary injury, iron dysregulation, neurovascular unit (NVU)

## Abstract

Stroke is a common and devastating disease with an escalating prevalence worldwide. The known secondary injuries after stroke include cell death, neuroinflammation, blood-brain barrier disruption, oxidative stress, iron dysregulation, and neurovascular unit dysfunction. Lipocalin-2 (LCN-2) is a neutrophil gelatinase-associated protein that influences diverse cellular processes during a stroke. The role of LCN-2 has been widely recognized in the peripheral system; however, recent findings have revealed that there are links between LCN-2 and secondary injury and diseases in the central nervous system. Novel roles of LCN-2 in neurons, microglia, astrocytes, and endothelial cells have also been demonstrated. Here, we review the evidence on the regulatory roles of LCN-2 in secondary injuries following a stroke from various perspectives and the pathological mechanisms involved in the modulation of stroke. Overall, our review suggests that LCN-2 is a promising target to promote a better understanding of the neuropathology of stroke.

## Introduction

Stroke is a common, destructive disease arising from vascular anomalies and has a high disability and mortality rate. Cerebral strokes can be classified into ischemic and hemorrhagic strokes. Changes in ischemic stroke caused by the loss of blood flow, glucose, and oxygen due to vascular obstruction, including triggering a series of oxidative, biochemical, and hormonal responses, ultimately lead to microvascular damage and blood-brain barrier (BBB) disruption. The mass effect of hematoma in hemorrhagic stroke and a series of intertwined degenerative cascades, including inflammation, red blood cell degradation, and iron deposition, and thrombin production, with the presence of some ischemic lesions distant from the ischemic focus and other pathophysiological mechanisms, such as oxidative stress and apoptosis, lead to the destruction of the BBB, cerebral edema, and hydrocephalus, among others, forming a vicious circle.

Lipocalin-2 (LCN-2), a 25 kDa protein, is involved in various biological reactions. As an immunomodulator, dysregulation of LCN-2 plays a vital role in several pathogeneses. Moreover, LCN-2 is involved in the pathophysiological processes of secondary injury after stroke. Here, we review the role of LCN-2 in pathophysiological processes, such as neuroinflammatory responses, dysregulation of intracellular iron levels and oxidative stress, and BBB and neurovascular unit (NVU) dysfunction. Additionally, we review the link between LCN-2 and cells. These findings contribute to a better understanding of the mechanisms underlying the involvement of LCN-2 in secondary injury after stroke, providing a potential target for stroke therapy.

## Origin, Structure, and Role of Lcn-2 in Cerebral Stroke

LCN-2 is a member of the lipocalin protein family. There are significant differences in the amino acid sequences of each member of the family, with <20% sequence homology between its members. LCN-2 has various biological functions. As an immunomodulator, alterations in LCN-2 levels are supposedly critical in many pathological processes. For instance, studies on humans indicated slightly elevated levels of LCN-2 in the plasma of patients with mild cognitive impairment (Choi et al., 2011) and the local concentration of LCN-2 in the brain tissue of patients with multiple sclerosis (Al Nimer et al., 2016). In a cerebral stroke, many cells express LCN-2. LCN-2 expression following ischemic stroke was reportedly increased both in the sera and brain, where it was localized to infiltrating neutrophils, cerebral endothelium, and a subset of astrocytes (Zamanian et al., 2012; Wang et al., 2015). LCN-2 expression was also found in astrocytes, microglia, neurons, and endothelial cells following intracerebral hemorrhage (ICH) in mice (Ni et al., 2015). However, LCN-2 is considered to be mainly expressed in astrocytes (Chia et al., 2011; Bi et al., 2013; Dong et al., 2013; Jin et al., 2014b; Table 1).

**Table 1:** Source of LCN-2 after stroke.

Ischaemia stroke	Infiltrating neutrophils, cerebral endothelium and a subset of astrocytes
Hemorrhage stroke	Astrocytes, microglia, neurons and endothelial cells

## Lcn-2 and Various Pathophysiological Process

### LCN-2 and NVU Dysfunction

NVU is defined by its function and anatomy (Harder et al., 2002) and is composed of endothelial cells, basement membrane, neurons, astrocytes, and pericytes (Amarenco et al., 2009; Abbott and Friedman, 2012; Gautam et al., 2020). It represents a conceptual framework that includes neurons and adjacent blood vessels (Iadecola, 2017). The interaction between the various components of NVU is extremely important and is gradually gaining attention (Lo and Rosenberg, 2009). The BBB and cerebral blood flow are precisely controlled by the NVU, thereby maintaining a homeostatic brain microenvironment (Armulik et al., 2010; Zlokovic, 2011). The disruption of the BBB, which is the core structure of the NVU, is an important part of early brain injury (Keep et al., 2018). In the study on the pathological mechanism of white matter damage caused by subarachnoid hemorrhage, LCN-2 was found to play an important role in the initiation and development of acute BBB disruption. LCN-2 deletion attenuates acute BBN leakage following subarachnoid hemorrhage (Egashira et al., 2016; Du et al., 2019). Another experiment showed that LCN-2 deficiency attenuated SAH-induced disruption of the white matter BBB, which further confirmed the effect of LCN-2 on the BBB in the opposite direction (Pang et al., 2017; Toyota et al., 2019). This dysfunction may be due to kainic acid-induced leakage of the BBB in the hippocampus (Shin et al., 2021).

**Figure 1:**
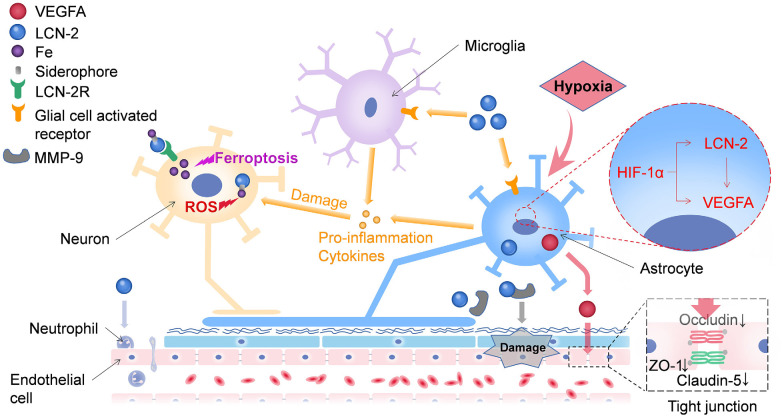
LCN-2 can damage neurons through multiple pathways. On one hand, Lipocalin-2 can transport iron into neurons by binding to LCN-2R, leading to the accumulation of intracellular iron, which in turn triggers ferroptosis and oxidative stress in neurons. On the other hand, LCN-2 can activate glial cells to release pro-inflammatory factors, which in turn damage neurons. LCN-2 has several ways to damage the BBB. First, it can promote the release of VEGFA from astrocytes, disrupt tight junctions between endothelial cells, and ultimately lead to leakage of the blood-brain barrier (BBB). Second, LCN-2 can bind to matrix metallopeptidase 9 (MMP-9), resulting in persistent BBB damage. Moreover, LCN-2 could induce neutrophil adhesion and damage the BBB. VEGFA, vascular endothelial growth factor A; LCN-2, lipocalin-2; LCN-2R, lipocalin-2 receptor; NVU, neurovascular unit; BBB, blood-brain barrier.

Several neutrophils infiltrate and damage the BBB (Wang Z. et al., 2020) during a stroke. Additionally, matrix metallopeptidase 9 (MMP-9) could be involved in the LCN-2-mediated BBB damage (Turner and Sharp, 2016; Figure 1). LCN-2 can combine with MMP-9 through disulfide bonds to reduce the degradation of MMP-9 and prolong its activity, thereby enhancing the damaging effect of MMP-9 on the BBB (Weng and Chou, 2015). Endothelial cells mainly constitute specialized membranes around blood vessels, and their damage can result in the destruction of the BBB (Armulik et al., 2010). Interestingly, LCN-2 may regulate endothelial cells in the BBB (Gasterich et al., 2021; Figure 1). LCN-2 induces the expression of vascular endothelial growth factor A (VEGFA), which affects vascular permeability either directly or *via* astrocytes (Kim et al., 2017). HIF-1α induces the expression of both LCN-2 and VEGFA in astrocytes. In LCN-2–/– mice, the hypoxia-induced expression of VEGFA is suppressed in the astrocytes, indicating that LCN-2 may be an upstream signaling factor for VEGFA. The upregulation of VEGFA reduces the levels of ZO-1, occludin, and claudin-5 and alters their distributions (Mondal et al., 2020; Wang G. et al., 2020; Yang et al., 2020), thereby increasing the permeability of the BBB and leading to abrogation of the NVU (Figure 1). As immune cells in the central nervous system (CNS), astrocytes can control the contraction and relaxation of pericytes and smooth muscle cells, thereby regulating cerebral blood flow (Janzer and Raff, 1987; Zonta et al., 2003). Microglia is not classically included as a part of the NVU; however, it is closely related to the structure and function of the NVU. They can modulate the innate immunity of astrocytes by releasing various signaling molecules (Kirkley et al., 2017; Liu L. R. et al., 2020). Studies have shown that LCN-2 can activate glial cells, release pro-inflammatory factors, and cause damage to neurons (Han et al., 2014). The chemokines released by LCN-2 after activating glial cells can also induce the infiltration of leukocytes (Tuttolomondo et al., 2014; Jayaraj et al., 2019), thus causing BBB leakage. Several red blood cells and damaged cells enter the brain tissue through the damaged BBB, further increasing the damage to neurons. Neurons play an important role in the NVU. Research has shown that LCN-2 can not only damage neurons through glial cells but can also directly trigger oxidative stress and apoptosis of nerve cells (Huang et al., 2020), which may be caused by the transportation of a large amount of iron into the cell (Shin et al., 2021). Therefore, the various components of the NVU are closely related in terms of structure and function to maintain the stability of brain functions. After a stroke, the damaging effect of LCN-2 renders the NVU susceptible to continuous damage, which in turn aggravates the secondary damage following the stroke.

### LCN-2 and Neuroinflammation

Neuroinflammation plays an important role in brain injury caused by hemorrhagic stroke (Wang and Doré, 2007). Studies have shown that LCN-2 is mainly expressed in astrocytes (Chia et al., 2011; Bi et al., 2013; Dong et al., 2013; Jin et al., 2014b) and is neurotoxic (Bi et al., 2013). However, other studies have found that inflammatory cells, such as microglia and neutrophils (Lee et al., 2007; Rathore et al., 2011; Jang et al., 2013b), can also express LCN-2. The effects of LCN-2 on microglia are complex. LCN-2 can increase the expression of M1-related genes in cultured mouse microglia. The expression of M1-related genes in microglia was significantly reduced in LCN-2-deficient mice, following lipopolysaccharide (LPS) injection (Jang et al., 2013b). LCN-2 expression is sensitive to cytotoxic agents, and inflammatory activation of microglia can lead to LCN-2 upregulation. Meanwhile, LCN-2 expression in BV-2 microglia induces changes in cell morphology. Microglial activation after ICH was weaker in LCN-2 knockout mice than in wild-type (WT) mice (Ni et al., 2015). This difference in microglial activation may contribute to differences in brain injury between WT and LCN-2 knockout mice after ICH.

In ischemic stroke, pro-inflammatory mediators regulated by LCN-2 play a key role in ischemia-reperfusion injury (Iadecola and Anrather, 2011). Afterastroke, iNOS expressed in microglia, astrocytes, endothelial cells, and infiltrating neutrophils releases large amounts of nitric oxide (NO; Iadecola and Anrather, 2011). NO reacts preferentially with reactive oxygen species (ROS) and forms peroxynitrite anion (ONNO^−^), which is a cytotoxic mitochondrial enzyme and genetic material. Inhibition of iNOS production by infiltrating neutrophils and brain endothelial cells provides prolonged neuroprotection after transient and permanent cerebral ischemia (Garcia-Bonilla et al., 2014). The level of interleukin-6 (IL-6), a pro-inflammatory cytokine, is elevated in plasma and brain 3–24 h after experimental stroke (Clark et al., 1999). Previous study results showed that *in vitro* exposure to IL-6 disrupted the integrity of the BBB by reducing the transendothelial electrical resistance (TEER) of the brain endothelial cells of rats (de Vries et al., 1996). CCL2 (monocyte chemoattractant protein 1, MCP-1) and CCL9 (macrophage inflammatory peptide gamma, MIP-1γ) are chemokines that are upregulated after ischemic stroke in humans (García-Berrocoso et al., 2014) and rodents (Shao et al., 2018). Furthermore, a previous study showed the involvement of CCL2 and its receptor “CCR2” in leukocyte trafficking after stroke (Conductier et al., 2010). Furthermore, studies have shown that genetic deletion of CCL2 (Hughes et al., 2002) and CCR2 (Dimitrijevic et al., 2007) reduces BBB permeability, accumulation of immune cells in ischemic brain tissue, and subsequent cerebral infarction (Chu et al., 2014). The role of CCL9 in stroke has not been investigated; however, deficiency of its receptor “CCR1” attenuates neutrophil adhesion to the vascular endothelium and migration to post-ischemic tissues (Reichel et al., 2006). In summary, these studies suggest that genetic or pharmacological inhibition of these pro-inflammatory mediators (iNOS, IL-6, CCL2, and CCL9) provides neuroprotection against stroke, which can affect pro-inflammatory mediators by modulating LCN-2.

Studies have shown that activated glial cells release cytokines and chemokines, which infiltrate leukocytes (Tuttolomondo et al., 2014; Jayaraj et al., 2019) and induce neuroinflammatory responses. Moreover, cytokines can damage neurons and destroy vascular and nerve coupling of the NVU (Figure 1). White blood cells can destroy the structure of the BBB, causing its leakage. Some studies have demonstrated that LCN-2 can promote the pro-inflammatory activation of glial cells and enhance the infiltration of neutrophils and macrophages into the brain under certain conditions.

### LCN-2 and Oxidative Stress

Oxidative stress plays a critical role in stroke (Rodrigo et al., 2013; Wang et al., 2018; Fumoto et al., 2019; Reiche et al., 2019). Moreover, LCN-2 plays an important role in oxidative stress. We reviewed the existing basic studies and found that few studies investigate the role of LCN-2 in oxidative stress following stroke; however, many conclusions have been drawn from oxidative stress after other nervous system diseases, which may indicate the direction for the study of oxidative stress following stroke with LCN-2. For example, in the experiment of nerve injury caused by KA, LCN-2 deficiency was found to reduce oxidative stress response (Shin et al., 2021). Leptin-deficient obese ob/ob mice show that LCN-2 is robustly induced in the hippocampus following obesity, and acts in an inflammatory manner by increasing BBB leakage and iron accumulation-induced oxidative stress (Jin et al., 2020). The mechanism underlying this phenomenon could involve the transformation of LCN-2 into an unfolded state by iron-loaded siderophores, leading to an increase in the intracellular levels of ROS (Huang et al., 2020; Figure 1). In an experiment using a mouse model of NASH, LCN-2 in the systemic upregulates the expression of the LCN-2 receptor (24p3R) in brain cells and secretes the damage-associated molecular pattern protein (DAMP), a high mobility group box 1 (HMGB1) that subsequently induces oxidative stress and nod-like receptor protein 3 (NLRP3) inflammasome activation on the brain cells (Mondal et al., 2020). Therefore, LCN-2 may promote oxidative stress and prevent oxidation. These findings suggest that LCN-2 may be used as a biomarker to identify oxidative stress. The protracted periods of oxidative stress and neuroinflammation provide an opportunity for therapeutic interventions. Immunotherapy designed to target pro-inflammatory mediators as a means of improving stroke outcome has, therefore, attracted considerable scientific attention (Yu et al., 2013; Lambertsen et al., 2019).

### LCN-2 and Iron Dysregulation

An increase in total iron content is observed in the lesion area in cases of both hemorrhagic and ischemic strokes (Tuo et al., 2017; Liu R. et al., 2020). The release of red blood cells primarily contributes to the presence of free iron after stroke. A large amount of free iron enters the brain parenchyma through the disrupted BBB and promotes ROS production by the Fenton reaction that induces oxidative stress and ferroptosis (Figure 1). Iron can also directly initiate toxic reactions, damage nerve cells, and cause NVU dysfunction (Righy et al., 2016; Liu J. et al., 2020). Furthermore, iron plays a major role in brain damage after ICH (Wagner et al., 2003; Xi et al., 2006). Brain non-heme iron increases after ICH in rats, and brain iron overload causes brain edema in the acute phase of ICH and brain atrophy thereafter (Xi et al., 2006; Keep et al., 2012). An iron chelator, deferoxamine, alleviates ICH-induced brain edema, neuronal death, brain atrophy, and neurologic deficits in rats and pigs (Xi et al., 2006; Keep et al., 2012; Xie et al., 2014). Clinical data also suggest that iron plays a role in ICH-induced brain injury. For example, clot lysis is associated with perihematomal edema development (Wu et al., 2006). Recent studies showed that high levels of serum ferritin, an iron storage protein, are independently associated with poor outcomes and severe brain edema in ICH patients (Mehdiratta et al., 2008; Pérez de la Ossa et al., 2010). LCN-2 is an acute-phase protein that is upregulated in inflammation, infection, and various injuries (Jha et al., 2015). It binds siderophores, which are secreted by microorganisms to scavenge iron (Goetz et al., 2002). However, evidence demonstrating the involvement of LCN-2 involved in iron homeostasis is increasing. A study showed that LCN-2 could be involved in cellular uptake or clearance of iron depending on iron status (Devireddy et al., 2005). Another report suggested that LCN-2 could mediate an alternative, transferrin-independent pathway for cellular iron delivery (Yang et al., 2002). In rats, LCN-2 is upregulated after ICH and may play a role in handling iron that is released from the hematoma during clot resolution (Dong et al., 2013). However, whether such a role is beneficial or detrimental is uncertain.

Overall, these results indicate that LCN-2 plays a role in iron-mediated brain injury after ICH. Until now, the detailed mechanism of iron delivery through LCN-2 has not been fully elucidated. In previous studies, LCN-2 was considered a mediator of an alternative, transferrin-independent pathway for cellular iron delivery (Yang et al., 2002). Iron is suggested to bind to an LCN-2-associated small molecular weight siderophore, transferred into cells through 24p3R, an LCN-2 cell-surface receptor, and subsequently released, resulting in an increased intracellular iron concentration (Flo et al., 2004; Devireddy et al., 2005). LCN-2 deficiency can block the pathway of LCN-2-dependent intracellular iron transportation, as suggested by the reduced iron-induced ferritin synthesis, and alleviate brain injury. However, studies showed that LCN-2 could regulate the intracellular iron concentration, and LCN-2 deficiency can increase the cellular iron levels in sepsis (Srinivasan et al., 2012). Thus, the role of LCN-2 in iron transport requires further study.

### LCN-2 and Brain Cell Death

Unlike other CNS diseases, a stroke leads to the death of numerous brain cells (Lee et al., 2007, 2012; Bi et al., 2013; Jang et al., 2013a; Jin et al., 2014a, b; Wang et al., 2015; Kim et al., 2016, 2017; Bhusal et al., 2019; Chen et al., 2019, 2020; Deng et al., 2019; Braga et al., 2020), which can be directly or indirectly mediated by LCN-2. As mentioned earlier, LCN-2 can activate glial cells to release pro-inflammatory factors that directly damage neurons. Additionally, LCN-2 can induce leukocyte infiltration as well as neuroinflammation by releasing chemokines following the activation of glial cells. LCN-2 can also directly trigger oxidative stress and apoptosis in neural cells. When LCN-2 is ectopically expressed by the 24p3R gene in iron-deficient cells, it further reduces intracellular iron levels. In cell types, such as astrocytes, neurons, and neural stem cells, cellular iron deprivation mediated by LCN-2 leads to apoptosis (Devireddy et al., 2001, 2005; Lee et al., 2009, 2012; Ferreira et al., 2018a). Furthermore, LCN-2 induces the expression of the proapoptotic protein Bim, which causes apoptosis (Devireddy et al., 2005; Lee et al., 2012). However, other studies indicate that Bim is not essential for LCN-2-mediated apoptosis (Lee et al., 2007; Naudé et al., 2012). LCN-2 can also directly initiate neuronal death *via* mitochondria-related pathways (Chen et al., 2020): this phenomenon is believed to occur through the mitochondrial apoptotic pathway (Iurlaro and Muñoz-Pinedo, 2016; Hetz and Papa, 2018). Endoplasmic reticulum stress-induced cell death occurs through the ATF4/CHOP or IRE1/JNK pathways. However, another study indicated that tunicamycin-induced LCN-2 cells produce a stronger endoplasmic reticulum stress response than WT cells. Moreover, LCN-2 acts as a protective factor and cells lacking LCN-2 are more prone to injury (Borkham-Kamphorst et al., 2020). Recombinant LCN-2 directly induces apoptosis in dopaminergic neurons in a dose-dependent manner (Weng et al., 2021). Additionally, LCN-2 can induce cell death by promoting the accumulation of intracellular iron (Xu et al., 2012; Ni et al., 2015; Dekens et al., 2018).

However, there are some controversies regarding the promotion of apoptosis by LCN-2 in brain cells. First, the idea that LCN-2 can directly initiate apoptosis is controversial. LCN-2 can initiate cell death *via* inflammation or cytotoxicity (Lee et al., 2007, 2009, 2012; Naudé et al., 2012; Mesquita et al., 2014). Moreover, many studies have shown that LCN-2 can significantly induce cytotoxicity (Bi et al., 2013; Wang et al., 2015; Kim et al., 2016, 2017). Second, the cell types in which LCN-2 can mediate toxicity are unclear, with different experiments producing different results. For example, a study found that LCN-2 affects the survivability of neurons (Bi et al., 2013), while other studies suggested that LCN-2 increases the sensitivity of astrocytes and microglia to cell death (Lee et al., 2007, 2009; Mesquita et al., 2014; Mike et al., 2019). The differences between these results may be due to variations in methodologies. For example, *in vivo* and *in vitro* experiments yield different results. Finally, while most of the studies emphasize the cytotoxic effects of LCN-2, high levels of LCN-2 may stimulate the glial cells to transform into cells that protect neurons (Xing et al., 2014). The high levels of LCN-2 may indicate SOS for the damaged neurons. For example, overexpression of LCN-2 reduces apoptosis in gastric mucosal cells (Wen et al., 2021). LCN-2 can prolong the survival of ovarian clear cell carcinoma cells by reducing iron-related oxidative stress (Yamada et al., 2016). When liver cells are stressed or damaged, LCN-2 protects them from apoptosis induced by endoplasmic reticulum stress (Borkham-Kamphorst et al., 2020). These observations may provide a new perspective on the role of LCN-2 in neurological diseases and help explore the mechanism underlying cell death after stroke.

## Lcn-2 Intervention Strategies

Studies have shown that neutralization of LCN-2 is a reasonable therapeutic strategy to alleviate reperfusion injury in stroke. Treatment with LCN-2 mAbs significantly attenuates LCN-2 mRNA and protein within a clinically relevant time window; however, targeting LCN-2 to inhibit post-stroke neuroinflammation may be more beneficial than inhibiting individual cytokines and chemokines, as LCN-2 may be responsible for the inflammatory cascade important upstream regulators of these mediators. Administration of LCN-2 mAb prior to full post-stroke LCN-2 elevation reduced the levels of LCN-2 and pro-inflammatory mediators (iNOS, IL-6, CCL2, and CCL9) and resulted in neutrophil infiltration, BBB leakage, cerebral infarction induction, and improved functional outcomes after stroke (Wang Z. et al., 2020). In addition to neutralizing LCN-2, other therapeutic approaches that inhibit the expression and secretion of LCN-2 (Cowland et al., 2006) or interfere with the interaction between LCN-2 and its receptors (Devireddy et al., 2005) are also potential avenues for therapeutic development (Suk, 2016).

## Perspective

There are some controversial results regarding the role of LCN-2 in some pathological processes (Table 2). Several studies have shown that LCN-2 could aggravate neuroinflammation (Lee et al., 2011; Jang et al., 2013a, b; Jin et al., 2014a); however, some studies found that neuroinflammation worsened in the absence of LCN-2 (Berard et al., 2012; Nam et al., 2014; Dekens et al., 2018; Kang et al., 2018). Additionally, other contradictory studies indicate that LCN-2 does not affect neuroinflammation (Ip et al., 2011; Lattke et al., 2017; Vichaya et al., 2019; Gasterich et al., 2021). Next, the effect of LCN-2 activation on cells was slightly different across various experiments. A recent study showed that LCN-2 triggered the classical activation of astrocytes in mice with tMCAO (Zhao et al., 2019), and another study found that LCN-2 can reduce inflammation in the astrocytes (Deng et al., 2019). However, Mike et al. found that LCN-2 does not affect the activation of astrocytes but influences the activation of microglia (Mike et al., 2019). Moreover, LCN-2 is crucial in the effects of PRX-2 on neutrophil infiltration and microglia/macrophage activation, and ultimately brain damage (Zhang et al., 2021). Thus, the contradictory conclusions may be due to differences in disease models, factors of induced inflammation, disease stages, and cell types. However, they all provide valuable contributions to the further understanding of post-stroke neuroinflammation.

**Table 2:** Controversial results of LCN-2 in some pathological processes.

LCN-2 and neuroinflammation	LCN-2 could cause neuroinflammation: Lee et al. (2011), Jang et al. (2013a, b), and Jang et al. (2014)
	The absence of LCN-2 could aggravate neuroinflammation: Berard et al. (2012), Nam et al. (2014), Dekens et al. (2018), and Kang et al. (2018)
	LCN-2 does not affect neuroinflammation: Ip et al. (2011), Lattke et al. (2017), Vichaya et al. (2019), and Gasterich et al. (2021)
LCN-2 and the activation of cells	LCN-2 could trigger the classical activation of astrocytes: Zhao et al. (2019)
	LCN-2 can reduce inflammation in the astrocytes: Deng et al. (2019)
	LCN-2 does not affect the activation of astrocytes but influences that of microglia: Mike et al. (2019)
	LCN-2 can affect neutrophil infiltration and microglia/macrophage activation: Zhang et al. (2021)
LCN-2 and oxidative stress	LCN-2 is able to promote oxidative stress: Huang et al. (2020), Jin et al. (2020), Mondal et al. (2020), and Shin et al. (2021)
	LCN-2 could reduce oxidative stress: Song et al. (2015), Xiao et al. (2016), Yamada et al. (2016), and Ferreira et al. (2018a)
LCN-2 and iron	LCN-2 contributes to iron accumulation: Dekens et al. (2018) and Shin et al. (2021)
	The absence of LCN-2 could cause iron accumulation: Nairz et al. (2009), (2015) and Ferreira et al. (2018a)

There are also some controversies regarding the role of LCN-2 in oxidative stress. In the conclusions mentioned earlier, LCN-2 is able to promote oxidative stress. However, one study found that the elevated expression of LCN-2 could reduce oxidative stress and the resulting cellular damage (Xiao et al., 2016; Yamada et al., 2016). In another study, knocking out the LCN-2 gene in mice increased oxidative stress, which could be attributed to an accumulation of active iron in neural stem cells (Ferreira et al., 2018a, b). Interestingly, LCN-2 can also exert antioxidant effects through several mechanisms. One mechanism may be that LCN-2 acts as an antioxidant by inducing the expression of heme oxygenase 1 (Song et al., 2015; Yamada et al., 2016). Another mechanism could be the overexpression of LCN-2 eliciting antioxidant effects by reducing the production of intracellular iron and protecting against ROS-induced oxidative stress (Xiao et al., 2016).

These controversial conclusions also exist in studies related to LCN-2 and iron. A study showed that LCN-2 contributes to iron accumulation (Shin et al., 2021). Another study validated the finding that a lack of LCN-2 significantly reduced Alzheimer’s disease-related hippocampal iron accumulation (Dekens et al., 2018). However, the absence of LCN-2 caused iron accumulation in certain cells, such as macrophages, hippocampal neurons, and neural stem cells (Nairz et al., 2009, 2015; Ferreira et al., 2018a). These controversial conclusions can provide new ideas for us to further explore the pathophysiological mechanism of LCN-2 in brain injury after stroke.

## Conclusion

Despite the abovementioned controversial conclusions, there is a consensus that LCN-2 can exacerbate brain injury. Several risk factors cause an accumulation of LCN-2 in the brain, which aggravates brain damage. Overall, it can be hypothesized that an increase in LCN-2 levels in response to injury aggravates the risk of poor outcomes in stroke, including causing inflammation in the brain, iron dysregulation, and neurovascular dysfunction. However, current direct evidence is insufficient. Therefore, future research should evaluate the direct link between LCN-2 and secondary stroke injuries. Additionally, studies show that LCN-2 plays contradictory roles, indicating that the function of LCN-2 is highly complex; thus, it is necessary to further explore its mechanism of action. The levels of LCN-2 may also be affected by multiple factors, including sex, age, disease type, and cell type. In summary, we need to further explore the role of LCN-2 in secondary brain injury after stroke and increase the usage of LCN-2 levels in the diagnosis and treatment of stroke.

## Author Contributions

All authors listed, have made substantial, direct and intellectual contribution to the work, and approved it for publication.

## Funding

This work was supported by grants from the National Natural Science Foundation of China (grant number 81560227) and the Yunnan Health Training Project of High-level Talents (grant number H-2017030).

## Conflict of Interest

The authors declare that the research was conducted in the absence of any commercial or financial relationships that could be construed as a potential conflict of interest.

## Publisher’s Note

All claims expressed in this article are solely those of the authors and do not necessarily represent those of their affiliated organizations, or those of the publisher, the editors and the reviewers. Any product that may be evaluated in this article, or claim that may be made by its manufacturer, is not guaranteed or endorsed by the publisher.
